# Urogenital Hiatus Closure: Facts, Fallacies, and Why a Unified Theory of Hiatal Failure is Needed

**DOI:** 10.1007/s00192-025-06153-7

**Published:** 2025-05-07

**Authors:** John O. L. DeLancey, James A. Ashton-Miller, Jennifer LaCross, Fernanda Pipitone, Payton Schmidt, Luyun Chen

**Affiliations:** 1 Department of Obstetrics and Gynecology, University of Michigan, 1500 E. Medical Center Dr., Ann Arbor, MI 48109 USA; 2 Department of Mechanical Engineering, University of Michigan, Ann Arbor, MI USA; 3 Hospital das Clinicas FMUSP, University of São Paulo, São Paulo, Brazil

**Keywords:** Hiatus, Levator ani, Pelvic floor, Pelvic organ prolapse, Perineal body, Perineal membrane

## Abstract

An enlarged urogenital hiatus is as important as apical support or fascial attachment failures in the development of prolapse and is strongly related to operative failure, yet we lack a conceptual model for factors responsible for hiatal failure. For a conceptual model to be valid, it cannot be proven false by empirical observation. We present six clinical observations with which future model development must be consistent. (1) Perineal body damage alone does not explain an enlarged urogenital hiatus. Three women have complete 4th degree lacerations but small hiatuses. (2) Levator damage is not a sole causal factor. One woman has bilateral levator avulsion but a normal hiatus, while another has intact muscles and an enlarged hiatus. (3) Hiatal assessment during straining is incomplete. Two women with similar straining urogenital hiatuses of 6–7 cm have respective 1.5 cm and 7 cm resting hiatuses. (4) Urogenital hiatus measurements during straining are confounded by Valsalva effort strength. Urogenital hiatus size increases by 30%, 51%, and 181% in one woman depending on straining strength. (5) Hiatal closure during pelvic muscle contraction differs widely. One woman can close her hiatus from 3.5 cm to 1.5 cm, while another shows no reduction despite evidence of contraction. (6) Prolapse/hiatus interactions occur with advancing age. One woman experiences progressive hiatal enlargement over 31 years. Our clinical observations reveal the complexity of the multiple factors involved in hiatal failure and support the need for a unified disease model consistent with these factors on which to base future research.

## Introduction

**Fig. 1:**
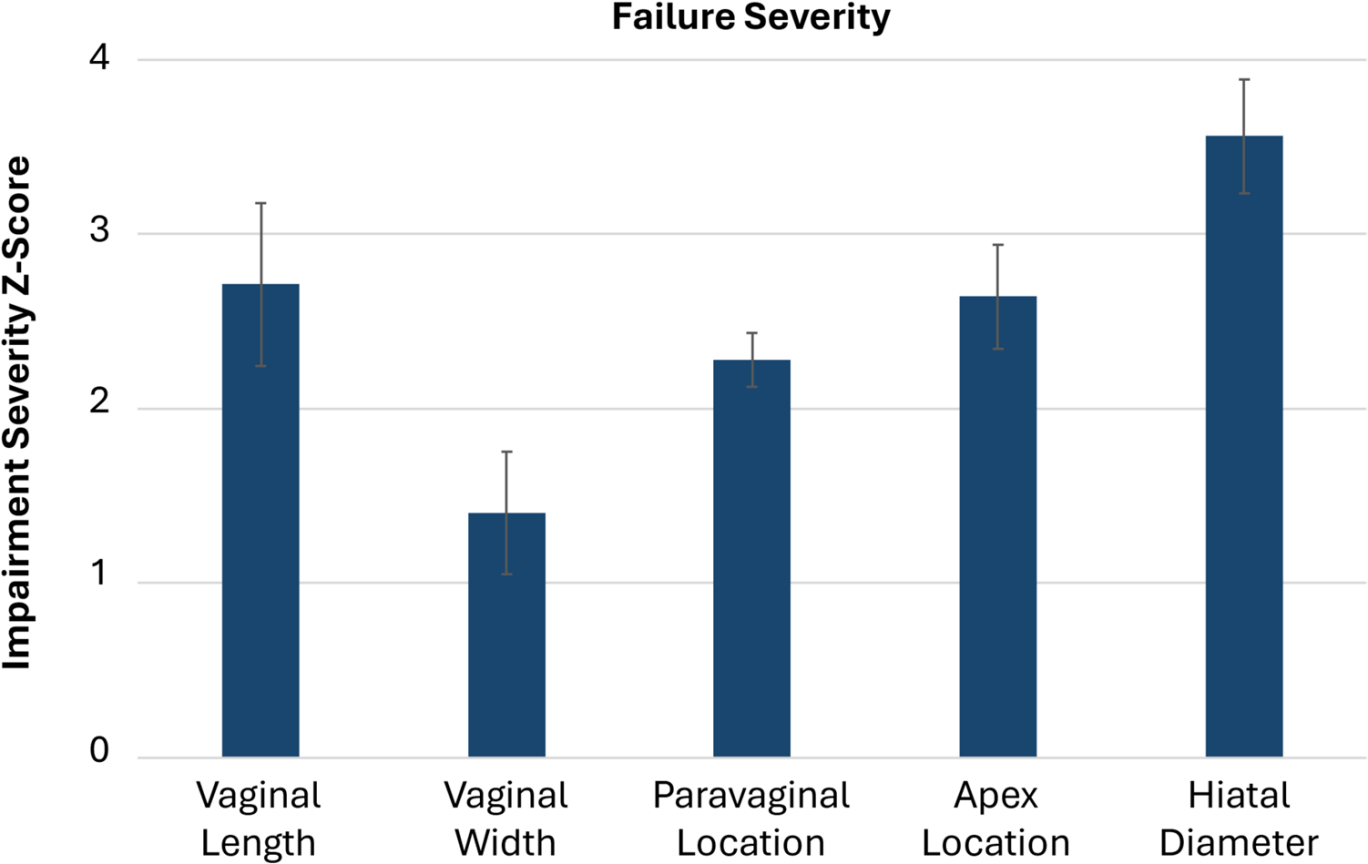
Failure severity in the five pelvic organ support system elements among women with prolapse

**Fig. 2:**
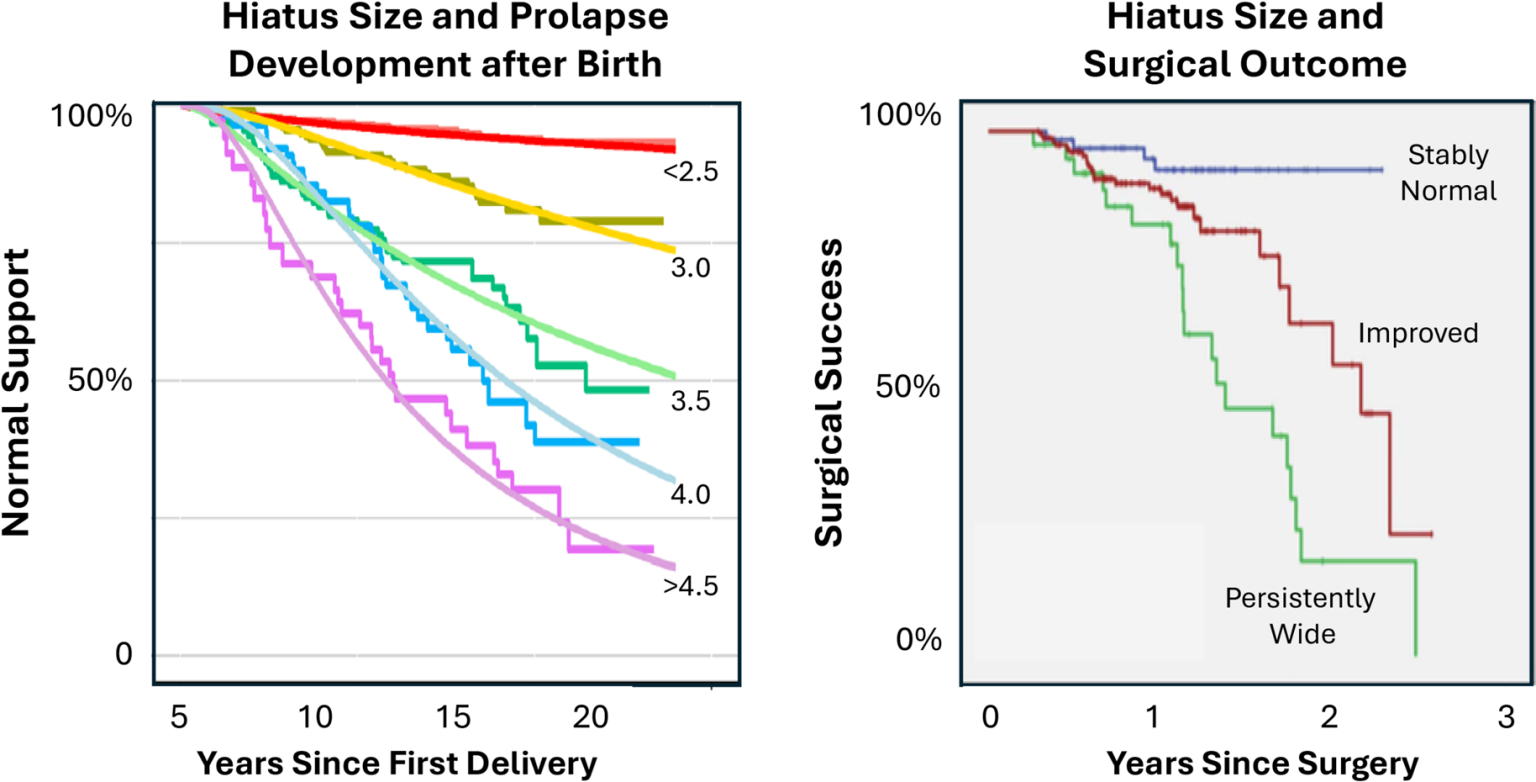
Hiatal size and its relationship to development of prolapse after birth and operative failure

There is strong scientific evidence from a properly controlled study that an enlarged urogenital hiatus that fails to close properly is as important to the development of pelvic organ prolapse as failure of any other pelvic floor support structure (Fig. 1) [1]. Indeed, an enlarged urogenital hiatus of ≥ 3.5 cm soon after birth is followed by a tenfold increase in the risk of developing prolapse over 10–15 years compared to those with a normal urogenital hiatus (Fig. 2) [2]. Furthermore, women with a persistently enlarged urogenital hiatus after surgery are six times more likely to have recurrent prolapse compared to those who have a normal urogenital hiatus before surgery [3]. The “thickness” of the perineal body is frequently discussed in this context with the idea that a “thin” perineal body in the presence of prolapse needs to be “built up” during surgery. This widely held, yet unfounded, idea inhibits progress toward a more complete understanding of why the urogenital hiatus fails to stay closed, what structural failures contribute to this problem, and how each might be addressed clinically.

A conceptual model that can guide and synthesize new research into the pathogenesis of the enlarged urogenital hiatus is needed. Although there is a considerable body of literature about changes in hiatal size [4], a lack of understanding about the anatomical apparatus that effects hiatal closure—and failures in individual elements of that apparatus that result in enlargement—impede progress. To help develop a coherent understanding of the *causes* of urogenital hiatal failure, a conceptual model must be consistent with clinical observations and emerging research findings.

We recently established the anatomical basis for such a model by defining the group of structures that surround the urogenital hiatus, which we refer to as the “perineal complex” [5]. This unit is like the rotator cuff in the shoulder, where several muscles and connective tissues work together to serve a common function. The perineal complex involves interconnected structures, including the medial levator ani, perineal membranes with their central connection through the perineal body, and the fascial tissues uniting these structures. This complex is more caudal and involves different structures than the more widely studied levator hiatus. We recently published a conceptual model for the entire pelvic floor [6]; however, it only includes the urogenital hiatus as one measurement element and does not address the underlying structural mechanics involved in urogenital hiatus structure and function.

Conceptual model development should be guided by the criterion for something to be considered “true”—that is, it cannot be proven false by empirical observation. To ensure development of such a model does not contain falsehoods based on what can be seen clinically, we have collected images of observations addressing six scenarios involving the urogenital hiatus. We herein present this photo essay to guide further consideration of the structural and functional factors associated with hiatal enlargement and development of a conceptual model that incorporates these factors.

### Perineal Body Damage Alone Does Not Result in an Enlarged Urogenital Hiatus

**Fig. 3:**
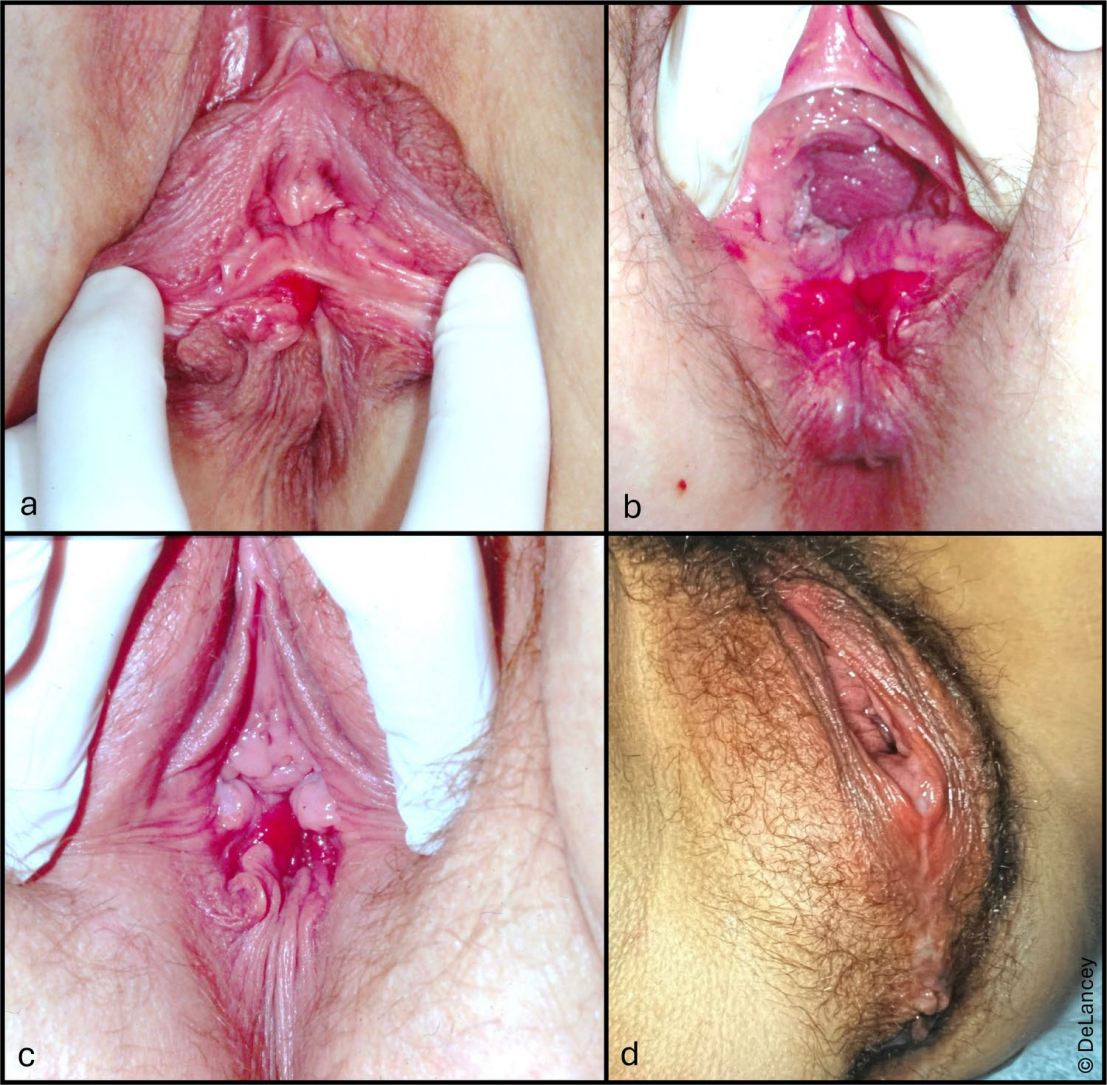
A closed hiatus in women with chronic 4 th degree lacerations but no prolapse (**a**–**c**) and a wide perineal body in a woman with prolapse (**d**)

The images in Fig. 3 address the hypothesis that the perineal body is *essential* to hiatal closure. Panels a, b, and c are women with chronic 4 th degree lacerations—none of whom had prolapse. The perineal body is completely missing, as indicated by the direct connection between the vaginal and rectal epithelia. Despite complete perineal body absence, the urogenital hiatuses remain tightly closed—likely due to the hypertrophy of the levator ani muscles. The distance between the urethra and the back of the anus is even less than the normal value of 5 cm [7], which can be judged by the 2 cm width of the examiner’s index finger. In fact, the image in panel b had to be taken during maximal strain to allow visualization of the relevant anatomy because the urogenital hiatus was so tightly closed that the junction between vagina and rectum could not be seen otherwise. Panel d shows a contrasting situation in a woman with an enlarged urogenital hiatus whose perineal body can be seen to be greatly elongated.

#### Implications

These observations are consistent with the finding that perineal body thickness and urogenital hiatus size are not linked, as has previously been documented [8]. Therefore, the hypothesis that the perineal body is a *major* determinant of hiatal closure can be rejected. This is not to say that connections between the two sides of the perineal complex through the perineal body region are not important, but perineal body damage alone is not sufficient to explain hiatal enlargement.

### Levator Ani Damage Alone Does Not Explain an Enlarged Urogenital Hiatus

**Fig. 4:**
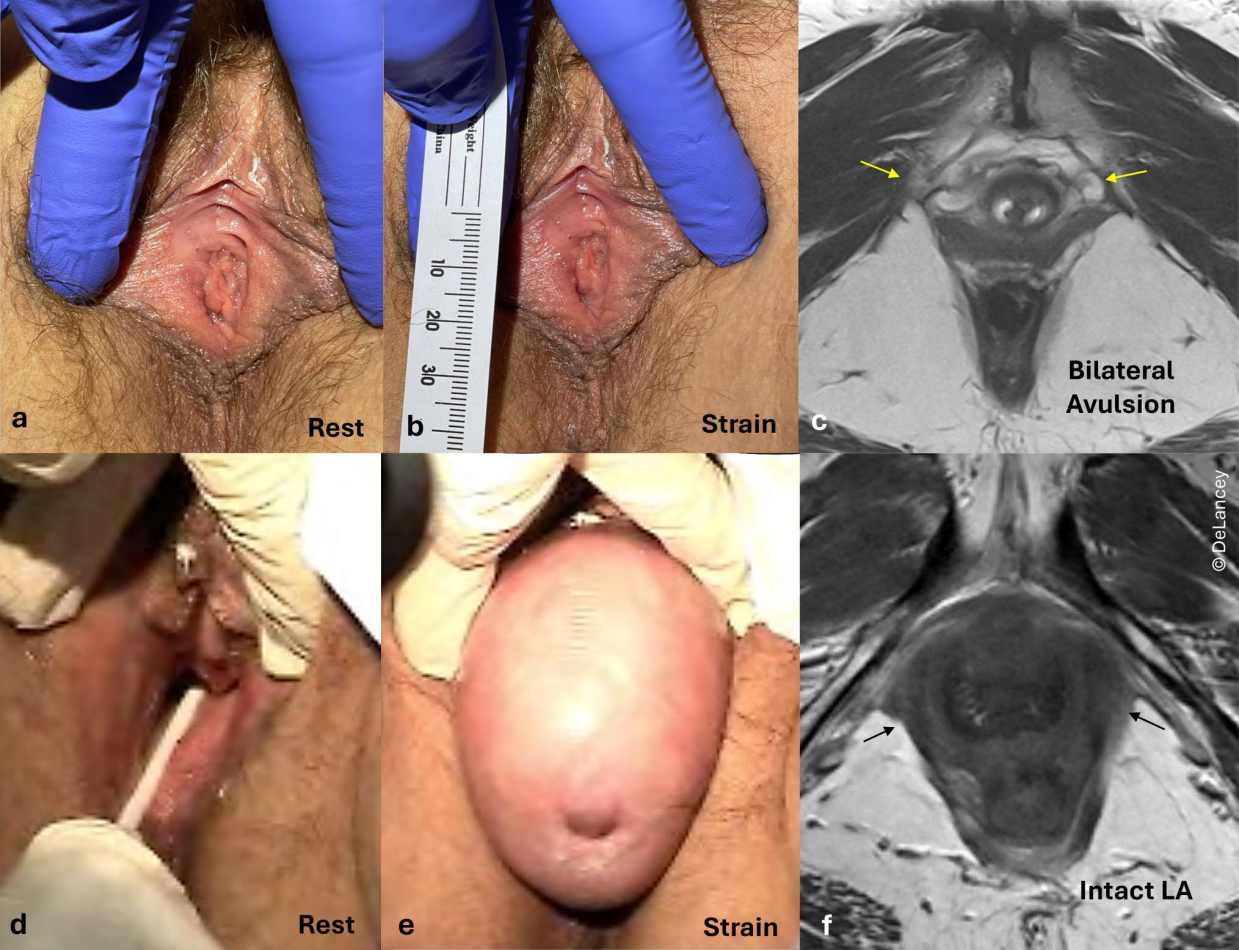
A woman with a normal hiatus during rest (**a**) and strain (**b**) with bilateral levator avulsion (**c**, yellow arrows) and a woman with an enlarged hiatus during rest (**d**) and strain (**e**) with intact levator muscles (**f**, black arrows)

Damage to the levator ani is clearly associated with an enlarged urogenital hiatus and pelvic organ prolapse [4]; however, it explains less than 20% of hiatal enlargement [9]. The examples shown in Fig. 4 present the paradox of a woman with a perfectly normal urogenital hiatus both at rest and at strain who has a bilateral avulsion seen on MRI versus a woman with a greatly enlarged urogenital hiatus and large prolapse with an intact levator ani.

#### Implications

This paradox illustrates that levator ani muscle avulsion, although clearly associated with pelvic organ prolapse, is alone not sufficient to explain hiatal enlargement. Instead, it must be considered in the wider context of failure of the other components in the perineal complex.

### Assessment of the Urogenital Hiatus Only During Straining is Incomplete

**Fig. 5:**
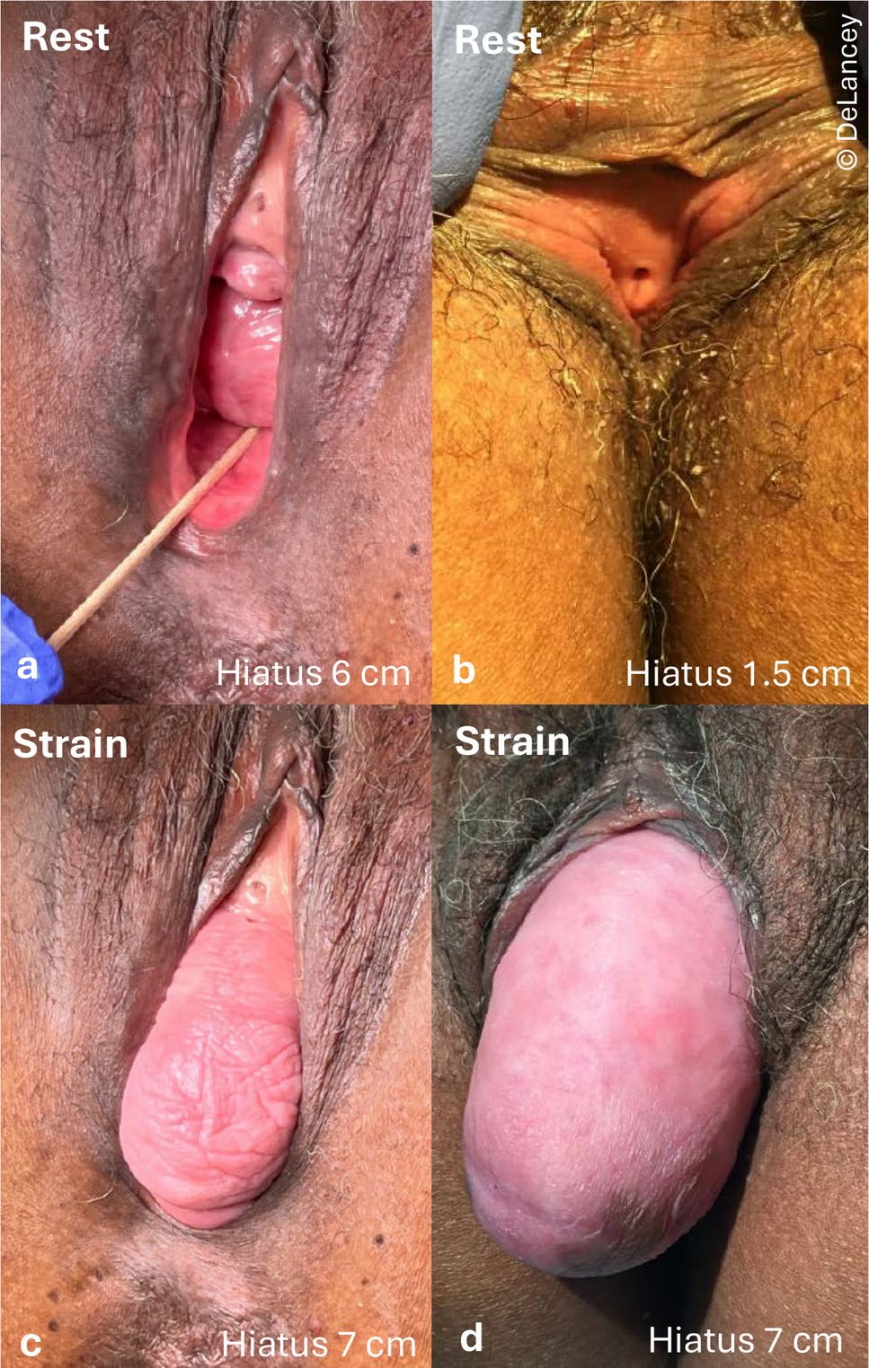
Two women with very different hiatus sizes at rest (**a**, **b**) contrasted with straining hiatus sizes that are similar in the same individuals (**c**, **d**)

Current Pelvic Organ Prolapse Quantification (POP-Q) evaluation only assesses the urogenital hiatus during straining. Rarely, if ever, do authors include data on the *resting* urogenital hiatus, or its width, which could represent a diastasis of the levator ani muscles. The images in Fig. 5 show the limitation of this convention, which may miss potentially valuable information. The top row shows two different individuals with markedly different hiatuses at rest: 6 cm vs. 1.5 cm—a 400% difference (panels a, b). However, during straining, the hiatuses are the same size. This illustrates the dilating effect of the prolapse on the urogenital hiatus during straining in certain women.

In addition, these images show that the *width* of the urogenital hiatus differs greatly between the two individuals—suggesting that the area of the urogenital hiatus is not just explained by an increase in anterior–posterior (A-P) diameter. In prior work, we showed that transverse diameter is responsible for about a quarter of area enlargement [8]. From a biomechanics standpoint, the force generated by abdominal pressure (recall that pressure is pounds *per square inch*) that drives the organs down is applied over the *area* of the urogenital hiatus—not just the A-P diameter. As an example, if the width in the first individual (panels a, c) is 4 cm, then the area is 6 cm × 4 cm = 24 cm^2^, while if the width in the second individual is 2 cm, the area is 1.5 cm × 2 cm = 3 cm^2^—an 800% difference, which is twice that seen from the difference in the A-P diameter alone. Width is easily estimated with two fingers plapating the medial margins of the levator ani, in the same way cervical dilation is assessed during labor [8]. This is relevant and calls attention to additional information that is not currently captured.

#### Implications

Because evaluating hiatal status only during straining is an incomplete characterization of hiatal status, measurements taken during rest might provide additional predictive information. The degree to which transverse diameter contributes to prolapse is yet to be determined. It would be appropriate to consider both transverse diameter and area in research studies, which has been done for the levator hiatus, but less so for the urogenital hiatus.

### Urogenital Hiatus Measurements are Confounded by Straining Effort

**Fig. 6:**
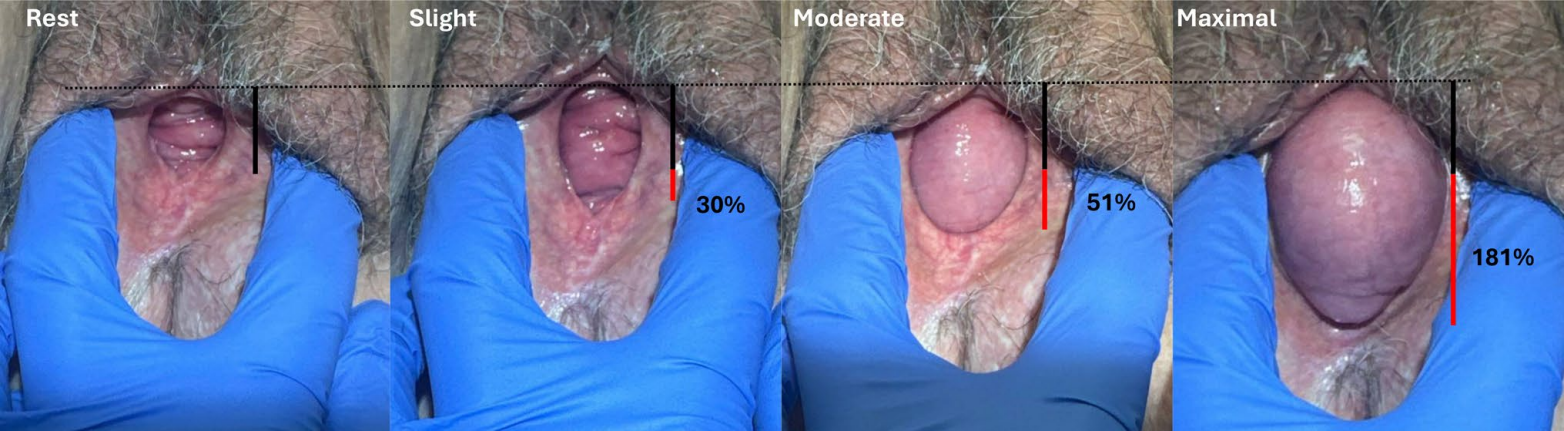
A woman with prolapse during different degrees of straining from rest to a maximal effort. The black vertical line indicates the size of the hiatus. The red line shows the difference from rest and percentage change from rest

The POP-Q records a single value for the A-P diameter of the urogenital hiatus. Clinicians are aware that prolapse size is affected by how hard and persistently a woman strains. The degree of this strain also affects hiatal size because the prolapse can push a normal urogenital hiatus open, as seen in Fig. 6. This issue is different than the comparison of *two different individuals* that was shown in Fig. 5, panel a. In this example of a *single individual*, compared to at rest, the urogenital hiatus is 30% larger with slight strain, 51% larger with moderate strain, and 180% larger with maximal strain.

#### Implications

Hiatal size during straining also depends on a patient’s Valsalva effort. Although it would be ideal to have all women strain to the same degree, this would limit straining to the pressure developed by the weakest individual. This example illustrates how the prolapse itself confounds assessment of hiatal size so that to some degree, the size of the urogenital hiatus is related to the size of the prolapse. This indicates the need for novel assessments that measure expansion of the urogenital hiatus with known dilating forces to assess resistance to hiatal opening independent of the presence and size of prolapse.

### Success and Failure of Urogenital Hiatus Normalization During Pelvic Muscle Contraction is Inconsistent

**Fig. 7:**
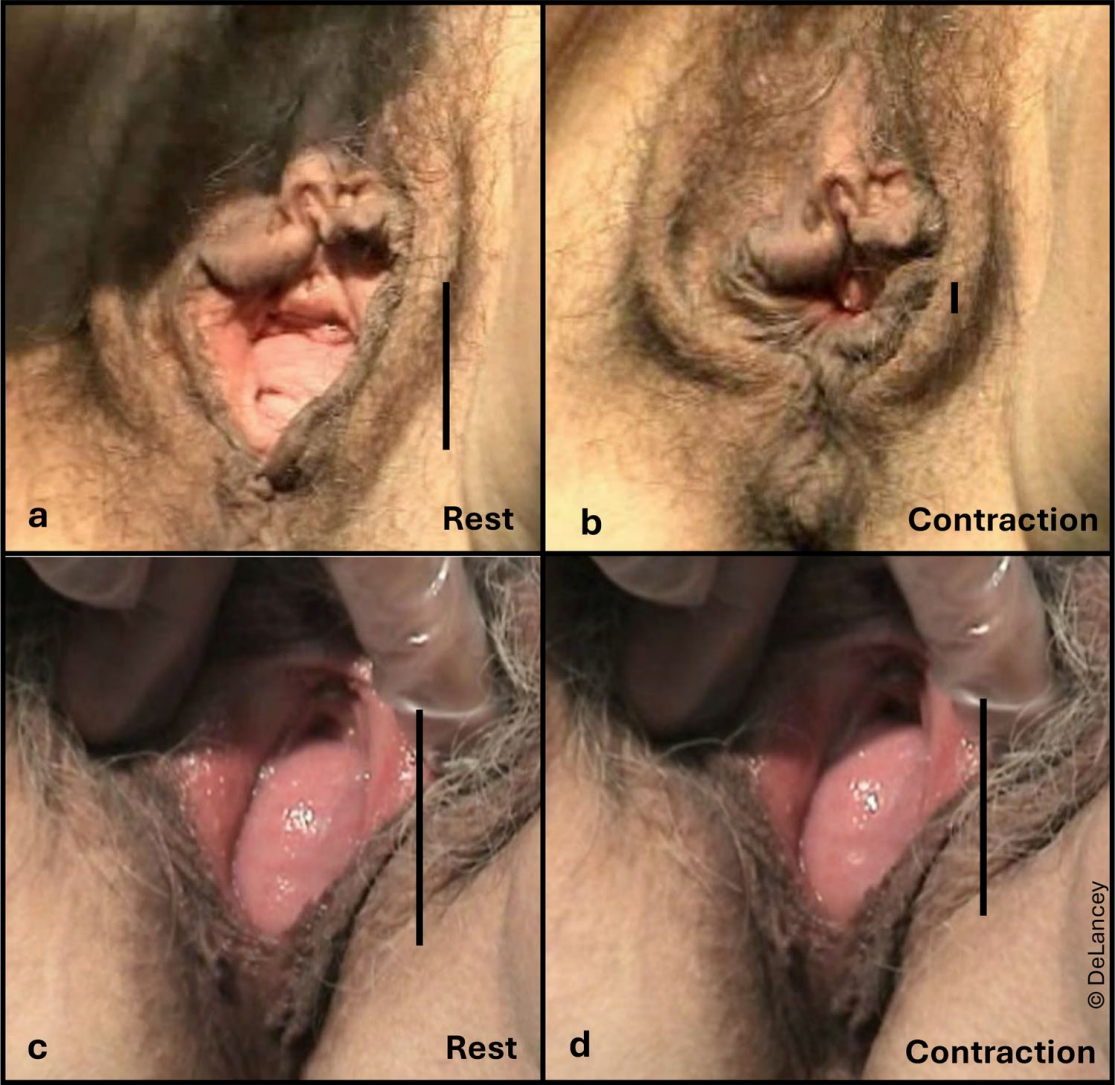
The different effects of pelvic muscle contraction on hiatal closure. Effective closure in one individual (**a**, **b**) does not occur in another (**c**, **d**), despite confirmation in the second individual that the anal sphincter and bulbospongiosus muscles contracted. The vertical bar indicates hiatus size

Figure 7 shows two women with an enlarged urogenital hiatus at rest and what happens with maximal contraction. The resting urogenital hiatus of the first woman (panels a, b) is returned to normal by a volitional pelvic muscle contraction, while that of the second woman is not (panels c, d)—despite confirmation that contraction occurred by observing tightening of the anal sphincter and contraction of the bulbospongiosus muscle. Similar to the muscles surrounding the spine, this pelvic floor muscle modifies its activity based on body position [10], postural perturbation [11], and the loads placed on it, including changes in intra-abdominal pressure [12, 13]. Normally there is greater activation of the levator ani when standing than when supine, and when the bladder is full. Motor control, including pelvic floor muscle activation timing and amplitude at rest and during activity, are the aspects of hiatal closure about which we have the least information. Studies of volitional activation are typically performed with little insight into automatic activation that occurs during daily tasks.

#### Implications

Efforts to understand the urogenital hiatus without evaluation of pelvic muscle motor control are incomplete. Unlike most skeletal muscles demonstrating silent electromyographic signals at rest, the levator ani muscle exhibits tonic activity without conscious effort of contraction, which increases with increased demand to maintain equilibrium. While there remains speculation about why an individual’s muscle can close the urogenital hiatus with contraction but does not do so at other times, specific research into its motor control lags behind that of its morphologic factors.

### Interaction Between Prolapse and the Urogenital Hiatus Over the Lifespan is Complex

**Fig. 8:**
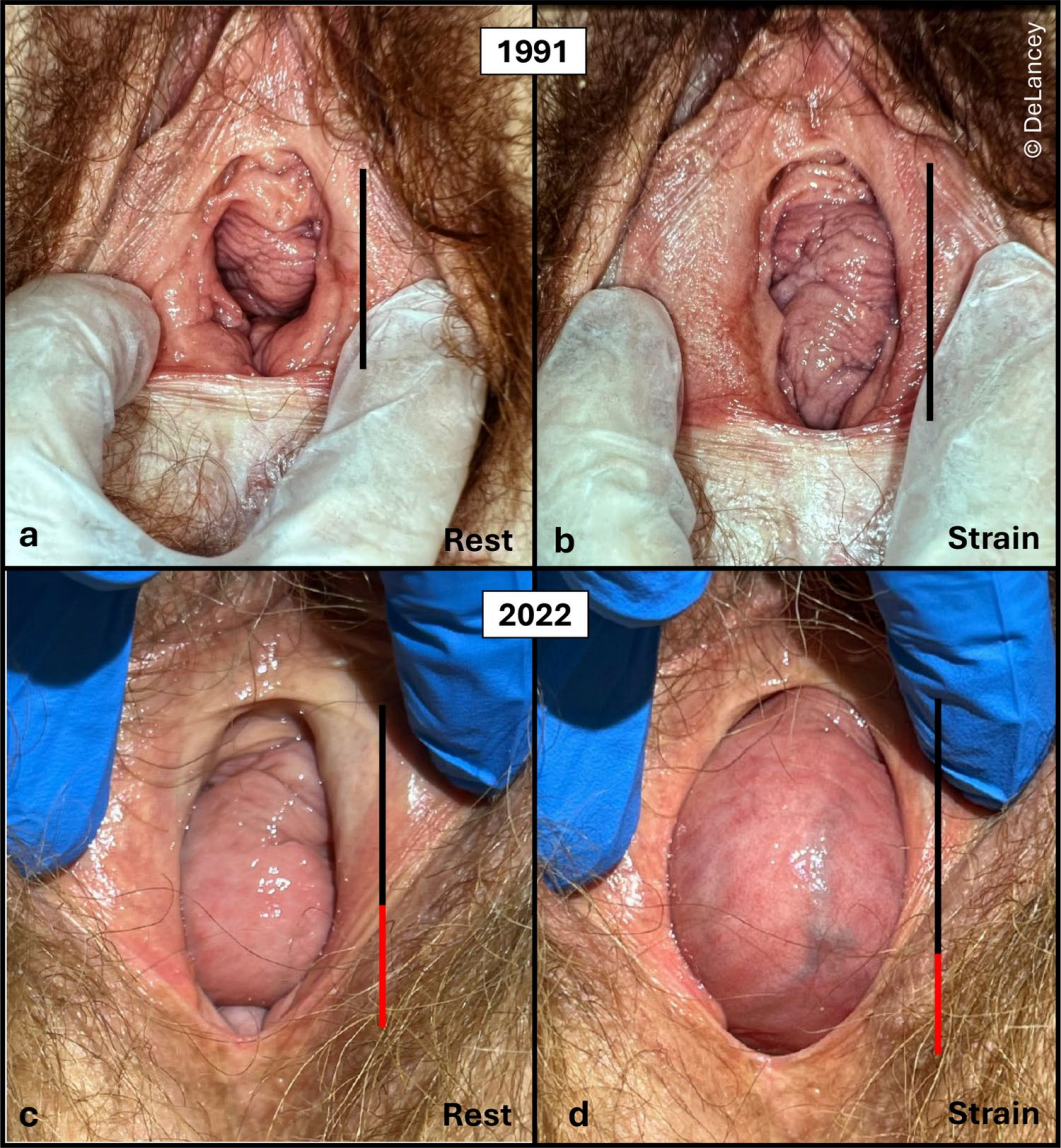
Hiatus at rest and strain in 1991 (**a**, **b**) compared to 2022 (**c**, **d**). The black line in a and b indicate hiatus size. In c and d, the 1991 dimension is shown in black and enlargement beyond that is indicated by red

In seeking to understand the factors involved in hiatal enlargement, stresses placed on the urogenital hiatus during a woman’s lifespan should be kept in mind. The images in Fig. 8 show urogenital hiatus changes over 31 years. Panels a and b show the urogenital hiatus at rest and strain in a woman in 1991, a year after delivering her fourth baby (weighing 10 lb. 14 oz.), while panels c and d show its status three decades later (2022). No intervening treatment was undertaken. Note that in 1991, the slightly enlarged urogenital hiatus is expanded by straining. In 2022, the resting urogenital hiatus A-P diameter is about 60% larger at rest and 40% larger during strain.

#### Implications

The urogenital hiatus is typically evaluated at one point in time during a woman’s lifespan, but aging effects need to be considered. There is a paradox between the progressive enlargement with advancing age seen in this figure and the small, tight urogenital hiatus found in many parous elderly women. So for the situation shown in the 1991 images with a slightly enlarged urogenital hiatus, the hiatus expands with advancing age (2022 images), but in other women without an injury, age does not result in hiatal enlargement [6]. This observation calls attention to our superficial understanding of the dynamic interplay between age-related changes and changes that occur with advancing age in women who sustained injury during birth. We hypothesize that an enlarged urogenital hiatus allows the vaginal walls to descend and further stretch the surrounding muscles and connective tissue due to repetitive loading. This is supported by the observation that some women using a pessary experience significant reduction of their urogenital hiatus size [14] when the dilating effect of the prolapse is eliminated.

## Synthesis and Call to Action

Given the importance of hiatal closure to pelvic organ prolapse and the limited understanding of the complex disease mechanisms illustrated by these clinical observations, there is need for an overall conceptual model that can synthesize these different findings. New investigative tools and studies to understand the several interacting factors involved are needed to understand urogenital hiatal closure mechanism failure. None of the individual scenarios illustrated above adequately capture the factors involved in hiatal enlargement, so it is necessary to determine how they interact.

The investigation of hiatal failure lags behind that of other injuries. For example, there are over 50 NIH-funded grants about the biomechanics and injury of the rotator cuff, but none concerned with the biomechanics of hiatal closure and the perineal complex—despite similar numbers of operations for the two conditions in women. This lag is likely due to the lack of background knowledge about musculoskeletal injury mechanisms among obstetricians and gynecologists, emphasizing the need for interdisciplinary research.

Given the importance of hiatal function and failure in the development of prolapse, a more complete analysis is needed so that prevention strategies and novel treatments can be targeted to the specific failure(s) present in each woman. New imaging strategies now allow each anatomical aspect of the perineal complex [15–18] to be evaluated. As was true when imaging first clearly documented anal sphincter injury during vaginal delivery [19] as an outcome variable, allowing risk factors to be established and different treatment and prevention strategies to be evaluated, specific experiments can be designed to establish the relative contribution of the several factors involved in hiatal enlargement and how they interact with one another.

There is a rich and unstudied biology of these tissues awaiting discovery research. With the ability to integrate the several aspects of hiatal structure and function, studies can be carried out to identify specific structural failures and their combinations so that targeted prevention and novel treatments can be developed and patient outcomes improved. As the eminent Swiss-American biologist Jean Agassiz observed, “Facts are stupid things until they are brought into harmony with a general law.” Hopefully, a new conceptual model, once developed and validated against clinical observations, can be formed to establish a unifying foundation for research into urogenital hiatus failure.
